# Threshold dynamics for a stage-structured model with non-local impulsive birth in a heterogeneous environment

**DOI:** 10.1007/s00285-026-02412-3

**Published:** 2026-05-21

**Authors:** Yurong Zhang, Taishan Yi, Yanyu Xiao

**Affiliations:** 1 School of Mathematical Sciences, Chongqing Normal University, Chongqing, 401331 China; 2 School of Mathematics (Zhuhai), Sun Yat-Sen University, Zhuhai, Guangdong, 519082 China; 3 Department of Mathematical Sciences, University of Cincinnati, OH, Cincinnati, 45221 USA

**Keywords:** Stage-structured population model, Reaction-advection-diffusion equation, Threshold dynamics, Heterogeneity, 35K57, 37N25, 92D25

## Abstract

In this paper, we examine the effect of spatial heterogeneity on a stage-structured population model with non-local impulsive birth. We begin by introducing a non-local impulsive reaction-advection-diffusion model with spatial heterogeneity to describe the stage-structured population dynamics, accounting for two distinct life cycle phases. Next, we convert this model into a discrete-time recursive system defined by a discrete map. By utilizing the theory of the asymptotic spectral radius of linearized operators, and verifying fundamental qualitative properties of the discrete map, we establish results on threshold dynamics of the recursive system, including the non-existence, existence, and global attractivity of non-trivial fixed points. Finally, numerical simulations are presented to illustrate the theoretical results and to assess the influence of key parameters, such as drift rates, mortality and reproduction functions, and dispersal kernels on population extinction or dispersal patterns.

## Introduction

The spatial spread of populations is a critical topic in ecology, epidemiology, and conservation biology, as it describes how organisms disperse across landscapes over time. Understanding the factors influencing population expansion is essential for managing invasive species, conserving endangered populations, and controlling the spread of structured population and infectious diseases. Mathematical models play a key role in studying population spread by incorporating biological processes such as birth, death, dispersal, and environmental heterogeneity. Under environmental pressures, some species exhibit active migration rather than random, diffusion-like movement. These dispersal mechanisms can be captured via various modeling approaches, including reaction-diffusion equations, integro-difference equations, and hybrid models that combine continuous and discrete-time dynamics. As a pioneer, genetic model was applied to invasive species spread by Skellam (1951), based on the work of Fisher (1937). Since then numerous spatially structured models based on reaction-diffusion equations have been developed over the past few decades (Cantrell and Cosner 2004; Murray 2002a, b). Berestycki and Hamel (2002); Berestycki et al. (2005); Xin (2000) focused on spatial heterogeneity and provided theoretical results and visualizations on population dispersal and persistent pattern. And Hu and Li (2015); Li and Otto (2024); Musgrave and Lutscher (2014a, 2014b) employed integro-difference equations and lattice dynamical systems to examine the population evolutions.

A common feature of the models in the studies mentioned above, as well as many subsequent works, is that while they account for spatial structure, they do not incorporate physiological structure. However, many species exhibit multiple distinct life stages, such as stoneflies, which have a complex life cycle with larval stages in river flows and adult winged stages on or near riverbanks. In 2016, Vasilyeva et al. (2016) introduced an impulsive reaction-diffusion model with a non-local impulse to describe population dynamics in streams, incorporating both aquatic larval and winged adult stages. More specifically, the partial differential equation models the drift of the larval stage, while the integral operator represents adult flight dispersal. The model is presented as follows,

1 $$\begin{aligned} \left\{ \begin{array}{ll} \frac{\partial u_n}{\partial t}(t,x)=d \frac{\partial ^2 u_n}{\partial x^2}(t,x)- q \frac{\partial u_n}{\partial x}(t,x) -f(u_n), & 0\le t\le \tau , \\ u_{n}(0,x)=u_{n,0}(x), & x\in \mathbb {R},\\ u_{n+1,0}(x) =g\big (\int _{\mathbb {R}} \mathcal {K}(x-y)u_n(\tau ,y) \textrm{d} y\big ), \end{array} \right. \end{aligned}$$

where $u_n(t,x)$ represents the larval population density at location *x* and time *t* during season *n*, and $\tau$ denotes a season length. The parameters *d* and *q* denote the diffusion and drift rates of larvae, respectively, while *f* accounts for density-dependent mortality, and *g* describes egg deposition and survival until the larval stage. In recent years, the impulsive differential equation has been widely used to study population dynamics (see (Bai et al. 2022; Fazly et al. 2017, 2020; Lewis and Li 2012; Lin and Wang 2015; Wang and Wang 2020; Wu and Zhao 2019, 2022; Zhang et al. 2024a)), and the impulsive harvesting has also been investigated (Meng et al. 2021, 2022). Additionally, Jin et al. (2016) extended the impulsive reaction-diffusion equation to model two-sex diffusing populations with short reproductive seasons. Wang et al. (2022) developed a stage-structured hybrid model, combining continuous and discrete time, to describe the spatio-temporal dynamics of species with highly structured life cycles. Bai et al. (2022) proposed a spatial population model by an impulsive reaction–diffusion equation and considered the synchronized emergence of mature individuals.

It is well known that spatial heterogeneity exerts a major influence on species distribution, ecological processes, and biodiversity (see (Alqawasmeh and Lutscher 2019; Cantrell and Cosner 1991; Latore et al. 1999; Lutscher et al. 2006, 2010)). To incorporate this influence on population density, we adopt a general function $f(x,\cdot )$ that depends on spatial variable *x* of arbitrary dimension *N* to capture the effects of spatial heterogeneity on stage-structured population dynamics. Additionally, we use a function $g(y,\cdot )$ to describe egg deposition and survival until the larval stage at location *y*. During the adult dispersal stage, we introduce the kernel function $\mathcal {K}$ to represent the flight dispersal of adult insects, where $\mathcal {K}(x-y)$ denotes the probability distribution of individuals moving from location *y* to *x*. The density of larvae in the next generation is given by the convolution,

$$\begin{aligned} \int _{\mathbb {R}^N} \mathcal {K}(x-y)g(y, u_n(\tau ,y)) \textrm{d} y. \end{aligned}$$

Based on this framework, we consider the following impulsive reaction-diffusion equation with spatial heterogeneity:

2 $$\begin{aligned} \left\{ \begin{array}{ll} \frac{\partial u_n}{\partial t} (t,x)=\nabla \cdot (D\nabla u_n(t,x)-q u_n (t,x)) +f(x,u_n(t,x)), & 0< t\le \tau , \\ u_{n}(0,x)=u_{n,0}(x), & x\in \mathbb {R}^N,\\ u_{n+1,0}(x) =\int _{\mathbb {R}^N} \mathcal {K}(x-y)g\big (y, u_n(\tau ,y)) \textrm{d} y, & x\in \mathbb {R}^N, \end{array} \right. \end{aligned}$$

where $q\in \mathbb {R}^N$, $D\in \mathbb {R}^{N\times N}$ is a constant, symmetric, positively defined matrix, $\nabla$ and $\nabla \cdot$ represent the gradient and divergence operators, respectively, and $\mathcal {K}$ is a nonnegative kernel satisfying $\int _{\mathbb {R}^N}\mathcal {K}(y)\textrm{d} y=1$ and $\mathcal {K}(0)> 0$. It is worth pointing out that this model extends and generalizes the work of Vasilyeva et al. (2016) by incorporating spatial heterogeneity into the model framework. In the presence of spatial heterogeneity, population dynamics is often studied under three typical habitat configurations: (i) a favorable habitat extends in one direction and an unfavorable habitat in the opposite direction (i.e., BG/GB-type); (ii) a habitat is favorable throughout the domain, except within a bounded region where conditions are unfavorable (i.e., GG-type); (iii) a bounded favorable region entirely surrounded by unfavorable habitat (i.e., BB-type). The researchers (Zhang et al. 2024a, b) studied *local impulsive* hybrid models within BG-type and GG-type shifting environments via propagation dynamics theory. However, the BB-type models are usually of analytical challenges and therefore received less attentions. Our work focuses on the threshold dynamics of a stage-structured population model incorporating *non-local impulsive* births in a BB-type environment.

This paper aims to investigate the threshold dynamics of a stage-structured population model with non-local impulsive birth and spatial heterogeneity, where a favorable habitat is bounded and surrounded by unfavorable regions. Notably, this impulsive reaction-advection-diffusion model can be reformulated as a discrete-time recursive system governed by a discrete map. By constructing an upper sequence of systems along with a generalized upper limiting system, we show that the solution of (2) asymptotically converges to zero at spatial infinity. By applying the Krein-Rutman theorem, the continuous dependence theorem, as well as Gelfand’s formula, we establish some properties of the asymptotic spectral radius $\rho _{\mathcal {L}}$, which is defined in (7). If $\rho _{\mathcal {L}}>1$, by constructing iterative inequalities and utilizing the comparison principle and threshold dynamics theory (Yi and Zhao 2023), we demonstrate the existence, uniqueness, and global attractivity of a positive fixed point. If $\rho _{\mathcal {L}}<1$, we apply the asymptotic spectral radius theory on amplification operator of the linearized system to exhibit the nonexistence of positive fixed points and the global attractivity of the trivial fixed point. In summary, we derive a threshold-type result using $\rho _{\mathcal {L}}$ as the threshold parameter.

The remainder of this paper is structured as follows. Section 2 introduces the necessary notations, assumptions, and fundamental results concerning on the properties of the solution map along with the reduction of the model to a discrete-time recursion governed by a discrete map. In Section 3, we analyze the threshold dynamics of (2). Section 4 presents the numerical simulations that support the theoretical findings. Finally, Section 5 summarizes the main results and provides further discussions.

## Preliminaries

Let *C* be the set of all bounded and continuous functions from $\mathbb {R}^N$ to $\mathbb {R}$ with the norm

$$\begin{aligned} \Vert \phi \Vert =\sum _{k=1}^\infty 2^{-k}\max \limits _{\Vert x\Vert _{\mathbb {R}^N}\le k}|\phi (x)| \text{ for } \phi \in C. \end{aligned}$$

Let $C_+=\{\phi \in C:\phi (x)\ge 0$ for $x\in \mathbb {R}^N\}$ and $C^\circ _+=\{\phi \in C_+:\phi (x)\in \textrm{Int} (\mathbb {R}^N_+)$ for $x\in \mathbb {R}^N\}$. Clearly, $C_+$ is a closed cone in *C*. For $\phi , \psi \in C$, we write $\phi \ge \psi$ if $\phi -\psi \in C_+$, $\phi >\psi$ if $\phi \ge \psi$ and $\phi \ne \psi$. For a given number $r>0$, we define $C_r:=\{\phi \in C:0\le \phi (x)\le r \text{ for } x\in \mathbb {R}^N\}$.

In the rest of this paper, unless stated otherwise, we assume that

**(F)**: $f, \partial _u f(\cdot ,\cdot )\in C(\mathbb {R}^N\times \mathbb {R}_{+},\mathbb {R})$, $f(x,0)=0$ for $x\in \mathbb {R}^N$ and there exist $K\in \mathbb {R}$ and $\tilde{N}>0$ such that $f(x,u)\le Ku$ for $x\in \mathbb {R}^N$ and $u\ge \tilde{N}$, $\frac{f(x,u)}{u}$ is nonincreasing in *u* for $x\in \mathbb {R}^N$, $\limsup \limits _{\Vert x\Vert \rightarrow \infty }\partial _u f(x,0)<\infty$ and $\inf \{\partial _u f(x,u):(x,u)\in \mathbb {R}^N\times [0,1]\}>-\infty$.

**(G)**: $g, \partial _u g(\cdot ,\cdot )\in C(\mathbb {R}^N\times \mathbb {R}_{+},\mathbb {R}_+)$, $g(x,\cdot )\in C^{1}(\mathbb {R}_{+},\mathbb {R}_+)$ and $g(x,0)=0$ for $x\in \mathbb {R}^N$, $g(\mathbb {R}^N\times (0,\infty ))\subseteq (0,\infty )$ and there exist $L>0$ and $\bar{N}>0$ such that $g(x,u)\le L\max \{ u,\bar{N}\}$ for $x\in \mathbb {R}^N$ and $u\in \mathbb {R}_+$. For any $x\in \mathbb {R}^N$, *g*(*x*, *u*) is nondecreasing in $u\in \mathbb {R}_+$ and $\frac{g(x,u)}{u}$ is nonincreasing in *u*, $\limsup \limits _{\Vert x\Vert \rightarrow \infty }\partial _u g(x,0)<\infty$.

It is well known that for any $\varphi \in C_+$, when $t\le \tau$ is not explicitly specified, the first equation of (2) with the initial value $u|_{t=0}=\varphi \in C_+$ admits a unique solution $u^\varphi (t,x;f)$ on $[0,\infty )$.

Now we introduce the following auxiliary equation:

3 $$\begin{aligned} \frac{ \partial u}{\partial t}(t,x)= \nabla \cdot (D\nabla u(t,x)-q u (t,x)) +f_+(u(t,x)),\;\;(t, x)\in (0,\infty )\times \mathbb {R}^N, \end{aligned}$$

where $$f_+\in C^1(\mathbb {R}_+,\mathbb {R})$$.

Let $\Phi [t,\varphi ;f],\Phi ^+[t,\varphi ;f_+]$ be the solutions of the first equation of (2) and equation (3) with the initial value $\varphi \in C_+$ when $t=0$. For simplicity, we denote $\Phi [t,\varphi ;f],\Phi ^+[t,\varphi ;f_+]$ by $\Phi [t,\varphi ],\Phi ^+[t,\varphi ]$ or $\Phi _{t}[\varphi ],\Phi ^+_{t}[\varphi ]$, respectively.

In the following, by virtue of (Zhang et al. 2025, Theorem 5.8), we establish some fundamental properties of $\Phi$ and $\Phi ^+$.

### Proposition 1

The following statements are valid.

(i) $\{\Phi _t\}_{t\ge 0}$ and $\{\Phi ^+_t\}_{t\ge 0}$ are two monotone, compact, and continuous semiflows on $C_+$. Moreover, $\Phi _t[\varphi ]\in C^\circ _+$ for $(t,\varphi )\in (0,\infty )\times (C_+\setminus \{0\})$. Here, we say a map $\Phi :\mathbb {R}_+\times C_+\rightarrow C_+$ is continuous (compact) if for any $t,r>0$, $\Phi |_{\mathbb {R}_+\times C_r}:\mathbb {R}_+\times C_r\rightarrow C_+(\Phi _t|_{C_r}: C_r\rightarrow C_+)$ is continuous (compact) in the usual sense.
(ii) If $f(z, \cdot )$ converges to $f_+$ in $C^1_{\textrm{loc}}(\mathbb {R}_+, \mathbb {R})$ as $z\rightarrow \infty$, then $\Phi ^+_{t}[\varphi ]=\lim \limits _{k,\Vert z\Vert \rightarrow \infty } \Phi _{t}[ T_{z}[\varphi _k]]( \cdot +z)$ in *C* with $\lim \limits _{k\rightarrow \infty }\Vert \varphi _k-\varphi \Vert =0$ for $(t,\varphi ,\varphi _k)\in \mathbb {R}_+\times C_+ \times C_+$, where $\sup \{|\varphi _k(x)|:x\in \mathbb {R}^N,k\in \mathbb {N}\}<\infty$.

According to the definition of $\Phi$, (2) can be reduced to a discrete-time recursion:

4 $$\begin{aligned} u_{n+1,0} = Q[u_{n,0}]:= \mathcal {K}*g(\cdot , \Phi _\tau [u_{n,0}])\qquad \text{ for } n\in \mathbb {N}, \end{aligned}$$

where $[\mathcal {K}*g(\cdot , u(\cdot ))](x)=\int _{\mathbb {R}^N} \mathcal {K}(x-y)g\big (y, u(y)) \textrm{d} y$ for $x\in \mathbb {R}^N$ and $u\in C_+$.

We now establish the positive invariance and compactness of *Q* based on the assumptions of *f* and *g*, as well as the definition of *Q*.

### Proposition 2

Let $Le^{K\tau }\le 1$. Then there exists a positive constant *M* such that for any $r>e^{|K|\tau }\max \{A_{K,M}, \bar{N},\tilde{N}\}$, we have $Q[C_{r}]\subseteq C_{r}$, and $Q[C_r]$ is precompact in *C*, where

5 $$\begin{aligned} A_{K,M} =\left\{ \begin{array}{ll} \frac{M}{K}, & K>0, \\ \frac{M(\tilde{N}+1)}{K+M}, & K\le 0. \end{array} \right. \end{aligned}$$

The above results are inspired by the proof techniques in (Zhang et al. 2024b, Lemma 3.1-(ii)). For completeness, detailed proofs are provided in the Appendix.

To ensure the positive invariance of *Q*, we always make the assumption that $Le^{K\tau }\le 1$ in the following analysis. The following results are concerned with strong positivity and continuity of *Q*.

### Proposition 3

Let *Q* be given in (4). Then, the following statements are true:

(i) $$Q[\varphi ]\in C^\circ _+$$ for $$\varphi \in C_+\backslash \{0\}$$.
(ii) If $f(z, \cdot )$ and $g(z, \cdot )$ converge in $C^1_{\textrm{loc}}(\mathbb {R}_+, \mathbb {R})$ to $f_+$ and $g_+$, respectively, as $z \rightarrow \infty$, then
  $$\begin{aligned} \lim \limits _{k,\Vert z\Vert \rightarrow \infty } Q[T_z[\varphi _k]]( \cdot +z)=Q_+[\varphi ]:=\mathcal {K}*g_+(\Phi ^+_\tau [\varphi ]) \end{aligned}$$
  in *C* with $\lim \limits _{k\rightarrow \infty }||\varphi _k-\varphi ||=0$ for $(\varphi ,\varphi _k)\in C_+ \times C_+$, where $\sup \{|\varphi _k(x)|:x\in \mathbb {R}^N,k\in \mathbb {N}\}<\infty$.

### Proof

(i) It follows from **(G)** that $g(\cdot ,\varphi (\cdot ))\in C_+\setminus \{0\}$ for $\varphi \in C_+\setminus \{0\}$. This, together with Proposition 1-(i) and the assumption of kernel function $\mathcal {K}$, yields $Q[\varphi ]=\mathcal {K}*g(\cdot ,\Phi _\tau [\varphi ](\cdot ))\in C^\circ _+$ for $\varphi \in C_+\setminus \{0\}$.

(ii) Let $G[\varphi ](x)=\mathcal {K}*g(\cdot ,\varphi (\cdot ))(x)$ for $x\in \mathbb {R}^N$ and $\varphi \in C_+$. Then $Q[\varphi ]=G\circ \Phi _\tau [\varphi ]$. According to Proposition 1-(ii) and (Yi and Zhao 2023, Proposition 4.4-(iv)), we see that

$$\begin{aligned} Q[T_z[\varphi _k]]( \cdot +z)= &  T_{-z}\circ Q\circ T_z[\varphi _k] \\= &  T_{-z}\circ G\circ \Phi _\tau \circ T_z[\varphi _k] \\= &  T_{-z}\circ G \circ T_z\circ T_{-z}\circ \Phi _\tau \circ T_z[\varphi _k] \\\rightarrow &  Q_+[\varphi ] \end{aligned}$$

as $k,\Vert z\Vert \rightarrow \infty$. This completes the proof. $\square$

To analyze the threshold dynamics of (4), we introduce the following assumption:

**(A)**: $$\limsup \limits _{\Vert x\Vert \rightarrow \infty } \partial _u g(x,0)e^{\limsup \limits _{\Vert x\Vert \rightarrow \infty } \partial _u f(x,0)\tau }<1$$.

From an ecological perspective, this condition corresponds to a BB-type habitat configuration, where the favorable habitat is spatially bounded and surrounded by unfavorable regions. It represents a phenomenon resulting from human constructions and vegetation degradations.

In order to analyze the threshold dynamics of the system (4), we next verify that *Q* meets the uniformly asymptotic annihilation **(UAA)** condition presented in Yi and Zhao (2023). A proof is given in the Appendix.

### Lemma 1

Let **(F)**, **(G)**, and **(A)** hold. Then there exists $$\widehat{Q}^+:C_+\rightarrow C_+$$ such that for any $$r^{**}>e^{|K|\tau }\max \{A_{K,M}, \bar{N},\tilde{N}\}$$, we have

(i) $$Q[\varphi ]\le Q[\psi ]\le Q[r^{**}]\le r^{**}$$ for $$0\le \varphi \le \psi \in C_{r^{**}}$$.
(ii) $$\lim \limits _{n\rightarrow \infty }|(\widehat{Q}^+)^n[r^{**}](0)|=0$$.
(iii) $\widehat{Q}^+$ is the generalized upper limiting system associated with *Q*, that is,
  $$\begin{aligned} \liminf \limits _{\Vert x\Vert \rightarrow \infty }\big [\inf \{(\widehat{Q}^+)^n[r^{**}](0)- T_{-x}\circ Q^n \circ T_x[r^{**}](0)\}\big ]\ge 0 \end{aligned}$$
  for $n\in \mathbb {N}$, where $A_{K,M}$ is given in (5).

Based on the above lemma, we obtain that the solution of the system asymptotically converges to zero at spatial infinity.

### Proposition 4

Let **(F)**, **(G)**, and **(A)** hold. Then

$$\begin{aligned} \lim \limits _{\alpha \rightarrow \infty }\big [\sup \{|N_n(x)|:(n,\Vert x\Vert )\in [\alpha ,\infty )^2 \}\big ]=0 \quad \text{ for } N_0=\varphi \in C_+. \end{aligned}$$

Hence, $\lim \limits _{n\rightarrow \infty }\Big [\sup \{ |N_n(x)|:\Vert x\Vert \ge n\varepsilon \}\Big ]=0$ for $\varphi \in C_+$ and $\varepsilon >0$.

### Proof

Fix $$\varphi \in C_+$$ and take $$r^{**}>1+\max \{\Vert \varphi \Vert _\infty ,e^{|K|\tau }\max \{A_{K,M}, \bar{N},\tilde{N}\}\}$$. Let

$$\begin{aligned} \overline{Q}[\psi ]=Q[\min \{\psi ,r^{**}-1\}],\quad \overline{Q}^+[\psi ]={\widehat{Q}}[\min \{\psi ,r^{**}-1\}] \end{aligned}$$

for $$\psi \in C_+$$, where $$\widehat{Q}$$ is defined as in the proof of Lemma 1. Then $$\overline{Q}[\psi ]\in C_{r^{**}-1}$$ for $$\psi \in C_+$$ due to Lemma 1-(i). Thus,

$$\begin{aligned} \limsup \limits _{n\rightarrow \infty }\Big [ \sup \{\frac{\overline{Q}^n[\psi ](x)}{r^{**}}:x\in \mathbb {R}^{N}\}\Big ]<1 \end{aligned}$$

for all $$\psi \in C_+$$. By Lemma 1 and the definition of $$\overline{Q}^+$$, we observe that $$(\overline{Q},\overline{Q}^+,r^{**})$$ satisfies the (**UAA**) as stated in Yi and Zhao (2023). Applying (Yi and Zhao 2023, Theorem 3.2) to $$\overline{Q}$$, we obtain

$$\begin{aligned} \lim \limits _{\alpha \rightarrow \infty }\big [\sup \{|\overline{Q}^n[\varphi ](x)|:(n,\Vert x\Vert )\in [\alpha ,\infty )^2 \}\big ]=0. \end{aligned}$$

Consequently, we derive the desired conclusion due to $N_n=Q^n[\varphi ]=\overline{Q}^n[\varphi ]$. $\square$

## Threshold dynamics

In this section, by using the theory of the asymptotic spectral radius of composite operators and applying the abstract theoretical framework, we investigate the threshold dynamics of system (2), including the existence, nonexistence, global attractivity, and uniqueness of nontrivial fixed points.

Following (Yi and Zhao 2023), we shall list the definitions of truncation, amplification, and asymptotic spectral radius, as well as the properties of the asymptotic spectral radius $\mathcal {L}$, where $\mathcal {L}:C \rightarrow C$ is a bounded, linear and positive operator on $(C,C_+,|\cdot |_{\infty })$.

For any $\varrho \in (0,\infty ]$, let

$$\begin{aligned} B_\varrho =\{x\in \mathbb {R}^{N}:\Vert x\Vert \le \varrho \},\qquad \zeta _{\varrho }(x):=\max \{0,\min \{1,\varrho -\Vert x\Vert \}\}, \forall x\in \mathbb {R}^{N}, \end{aligned}$$

where $$B_\infty $$ represents $$\mathbb {R}^{N}$$.

Define the truncation operator $\mathcal {L}_\varrho :C(B_\varrho ,\mathbb {R})\rightarrow C(B_\varrho ,\mathbb {R})$ by $\mathcal {L}_\varrho [\varphi ]=\mathcal {L}[\zeta _\varrho \varphi ]|_{B_\varrho }$, where $(\varrho ,\varphi )\in (0,\infty ]\times C(B_\varrho ,\mathbb {R})$ and

6 $$\begin{aligned} (\zeta _\varrho \varphi )(x)=\left\{ \begin{array}{ll} \zeta _\varrho (x) \varphi (x), & x\in B_\varrho , \\ 0, \qquad & x\notin B_\varrho . \end{array} \right. \end{aligned}$$

Define

7 $$\begin{aligned} \rho _\mathcal {L}:=\lim \limits _{\varrho \rightarrow \infty }\rho (\mathcal {L}_\varrho ), \end{aligned}$$

where $\rho (\mathcal {L}_\varrho )$ represents the spectral radius of $\mathcal {L}_\varrho$. In Yi and Zhao (2023), $\rho _{\mathcal {L}}$ is called the *asymptotic spectral radius* of $\mathcal {L}$.

For any given $(\sigma ,d,\varrho )\in Int(\mathbb {R}_+^2) \times (0,\infty ]$ and $(\varphi ,\psi )\in C\times C(B_\varrho ,\mathbb {R})$, define the amplification operator $\widehat{\mathcal {L}}_{\sigma ,d}:C\rightarrow C$ by

$$\begin{aligned} \widehat{\mathcal {L}}_{\sigma ,d}[\varphi ](x)=(1+\sigma \zeta _{d}(x))\mathcal {L}[\varphi ](x), \, \forall x\in \mathbb {R}^{N}, \end{aligned}$$

and $$\widehat{\mathcal {L}}_{\sigma ,d,\varrho }:C(B_\varrho ,\mathbb {R})\rightarrow C(B_\varrho ,\mathbb {R})$$ by $$\widehat{\mathcal {L}}_{\sigma ,d,\varrho }[\psi ]=\widehat{\mathcal {L}}_{\sigma ,d}[\zeta _\varrho \psi ]|_{ B_\varrho }$$.

Let $\mathbb {L}[t,\varphi ;\xi ]$ be the solution of the following linear equation:

$$\begin{aligned} \left\{ \begin{array}{ll} \frac{\partial u}{\partial t} (t,x)= \nabla \cdot (D\nabla u(t,x)-q u (t,x)) +\xi (x)u(t,x), & t>0, \\ u(0,x)=\varphi (x), & x\in \mathbb {R}^N, \end{array} \right. \end{aligned}$$

where $\xi ,\varphi \in C$. For any $t\in \mathbb {R}_+$ and $\xi \in C$, we further define

$$\begin{aligned} \mathbb {L}_+[t,\cdot ;\xi ] =\mathbb {L}[t,\cdot ;\limsup \limits _{\Vert x\Vert \rightarrow \infty }\xi (x)]. \end{aligned}$$

By virtue of (Zhang et al. 2025, Theorem 5.8), we can readily obtain the following results.

### Lemma 2

Let $\xi \in C$. Then the following statements are valid:

(i) $\mathbb {L}[t,\cdot ;\xi ], \mathbb {L}_+[t,\cdot ;\xi ]: C \rightarrow C$ are bounded and positive linear operators on $(C,C_+,|\cdot |_{\infty })$ for each $t\in \mathbb {R}_+$, and $\mathbb {L}[t,\cdot ;\xi ], \mathbb {L}_+[t,\cdot ;\xi ]:C_r\rightarrow C$ are continuous and compact operators for each $(r,t)\in (0,\infty )^2$.
(ii) If $\lim \limits _{\Vert x\Vert \rightarrow \infty }\xi (x)=\xi (\infty )$. Then $\lim \limits _{k,\Vert x\Vert \rightarrow \infty } \Vert T_{-x}\circ \mathbb {L}[t,\cdot ;\xi ]\circ T_x[\varphi _k] \Vert =\mathbb {L}_+[t,\varphi ;\xi ]$ provided that $\lim \limits _{k\rightarrow \infty }\Vert \varphi _k-\varphi \Vert =0$, where $(t,\varphi _k,\varphi )\in \mathbb {R}_+\times C_+ \times C_+$ and $\sup \{|\varphi _k(x)|:x\in \mathbb {R}^N,k\in \mathbb {N}\}<\infty$.

Assume $\theta \in C$ and $\eta \in C_+\backslash \{0\}$. Let $\mathbb {G}[\varphi ](x)=\mathcal {K} * [\eta (\cdot )\varphi (\cdot )](x)$ for $x\in \mathbb {R}^N$ and $\varphi \in C_+$, and denote $\mathcal {L}[\cdot ;\eta ,\theta ]=\mathbb {G}\circ \mathbb {L}[\tau ,\cdot ;\theta ]$. Then we have the following observation.

### Lemma 3

Let $\mathcal {L}:=\mathcal {L}[\cdot ;\eta ,\theta ]$ and $\mathcal {L}_+=\mathcal {L} [\cdot ;\limsup \limits _{\Vert x\Vert \rightarrow \infty }\eta (x),\limsup \limits _{\Vert x\Vert \rightarrow \infty }\theta (x)])$. Then the following statements are valid.

(i) $\mathcal {L}$ and $\mathcal {L}_+$ are bounded, linear and positive operators on $(C,C_+,|\cdot |_{\infty })$, and $\mathcal {L}, \mathcal {L}_+:C_r \rightarrow C$ are continuous and compact maps for each $r>0$.
(ii) If $\limsup \limits _{\Vert x\Vert \rightarrow \infty }\eta (x)e^{\limsup \limits _{\Vert x\Vert \rightarrow \infty }\theta (x)\tau }<1$, then $\mathcal {L}$ satisfies the asymptotic contraction assumption **(AC)** as stated in Yi and Zhao (2023).

The detailed proofs of the properties of $\mathcal {L}$ and $\mathcal {L}_+$ are provided in the Appendix.

In view of (Yi and Zhao 2023, Lemma 4.1 and Lemma 4.12), we present the following results that provide lower bounds for the functions *f* and *g*.

### Lemma 4

For any $\delta ,\iota >0$, there exists $\xi ^{*}=\xi ^{*}_{ \delta ,\iota }>0$ such that $f(x,u)\ge (\partial _u f(x,0)-\delta )u$ for $(x,u)\in B_{\iota +1}\times [0,\xi ^{*}]$. Let $\mu ^*=-\inf \{\partial _u f(x,u):(x,u)\in \mathbb {R}^N\times [0,1]\}<\infty$. Then $f(x,u)\ge \Gamma _{\theta , \delta ,\iota }(x)u$ for $(x,u)\in \mathbb {R}^{N} \times [0,\xi ^{*}]$, where $\theta =\partial _u f(\cdot ,0)$ and $\Gamma _{\theta , \delta ,\iota }\in C$ is defined by

$$ \Gamma _{\theta , \delta ,\iota }(x)=\left\{ \begin{array}{ll} -\mu ^*+\max \{0,\theta (x)-\delta +\mu ^*\}, & x\in B_{\iota }, \\ -\mu ^*+\max \{0,\theta (x)-\delta +\mu ^*\}(1+\iota -\Vert x\Vert ), \qquad & x\in B_{\iota +1}\setminus B_{\iota }, \\ -\mu ^*, \qquad & x\notin B_{\iota +1}. \end{array} \right. $$

### Lemma 5

For any $\delta ,\iota >0$, there exists $\xi ^{*}=\xi ^{*}_{ \delta ,\iota }>0$ such that $g(x,u)\ge (\partial _u g(x,0)-\delta )u$ for all $(x,u)\in B_{\iota +1}\times [0,\xi ^{*}]$. Hence, $g(x,u)\ge \Lambda _{\eta , \delta ,\iota }(x)u\ge 0$ for all $(x,u)\in \mathbb {R}^{N} \times [0,\xi ^{*}]$, where $\eta =\partial _u g(\cdot ,0)$ and $\Lambda _{\eta , \delta ,\iota }\in C(\mathbb {R}^{N},\mathbb {R}_+)$ is defined by

$$ \Lambda _{\eta , \delta ,\iota }(x)=\left\{ \begin{array}{ll} \max \{0,\eta (x)-\delta \}, & x\in B_{\iota }, \\ \max \{0,\eta (x)-\delta \}(1+\iota -\Vert x\Vert ), \qquad & x\in B_{\iota +1}\setminus B_{\iota }, \\ 0, \qquad & x\notin B_{\iota +1}. \end{array} \right. $$

As introduced in the above lemmas, we set $\theta =\partial _u f(\cdot ,0)$ and $\eta =\partial _u g(\cdot ,0)$, and will continue to use this convention in what follows.

### Lemma 6

If $\rho _{\mathcal {L}}>1$, then there exists $\delta _0>0$ such that $\rho _{\underline{\mathcal {L}}}>1$, where $\underline{\mathcal {L}}=\mathcal {L}[\cdot ; \Lambda _{\eta , \delta _0,\frac{1}{\delta _0}},\Gamma _{\theta , \delta _0,\frac{1}{\delta _0}}]$.

### Proof

By the definition of $\rho _{\mathcal {L}}$, we observe that there exists $\iota _0>0$ such that $\rho (\mathcal {L}_{\iota _0})>1$. This, combined with the compactness of $\mathcal {L}_{\iota _0}$ and (Zeidler 1986, Proposition 7.26), implies that $\mathcal {L}_{\iota _0}[\psi _0]=\rho (\mathcal {L}_{\iota _0})\psi _0$ for some $\psi _0\in C(B_{\iota _0},(0,1])$. Define

$$\begin{aligned} \mathbb {G}^\delta [\varphi ](x):=\mathcal {K} * [\Lambda _{\eta , \delta ,\frac{1}{\delta }} (\cdot )\varphi (\cdot )](x),\quad x\in \mathbb {R}^N \text{ and } \varphi \in C_+, \end{aligned}$$

where $\Lambda _{\eta , \delta ,\frac{1}{\delta }}(\cdot )$ is defined in Lemma 5. Then $\lim \limits _{\delta \rightarrow 0,k\rightarrow \infty }\mathbb {G}^\delta [\varphi _k]\rightarrow \mathbb {G}[\varphi ]$ in *C* with $\lim \limits _{k\rightarrow \infty }\Vert \varphi _k-\varphi \Vert =0$ for $(\varphi ,\varphi _k)\in C_+ \times C_+$, where $\sup \{|\varphi _k(x)|:x\in \mathbb {R}^N,k\in \mathbb {N}\}<\infty$.

Set $\mathcal {L}^\delta :=\mathcal {L}[\cdot ; \Lambda _{\eta , \delta ,\frac{1}{\delta }},\Gamma _{\theta , \delta ,\frac{1}{\delta }}]=\mathbb {G}^\delta \circ \mathbb {L}[\tau ; \cdot ,\Gamma _{\theta , \delta ,\frac{1}{\delta }}]$, where $\Gamma _{\theta , \delta ,\frac{1}{\delta }}(\cdot )$ is defined in Lemma 4. Since $\Gamma _{\theta , \delta ,\frac{1}{\delta }}\rightarrow \theta$ in *C* as $\delta \rightarrow 0$ and by the theorem of continuous dependence Zhang et al. 2025, Theorem 5.8, it follows that $\mathbb {L}[\tau ; \zeta _{\iota _0}\psi _0,\Gamma _{\theta , \delta ,\frac{1}{\delta }}]\rightarrow \mathbb {L}[\tau ; \zeta _{\iota _0}\psi _0,\theta ]$, and hence,

$$\begin{aligned} \mathcal {L}^\delta [\zeta _{\iota _0}\psi _0] =\mathbb {G}^\delta \circ \mathbb {L}[\tau ; \zeta _{\iota _0}\psi _0,\Gamma _{\theta , \delta ,\frac{1}{\delta }}] \rightarrow \mathbb {G}\circ \mathbb {L}[\tau ; \zeta _{\iota _0}\psi _0,\theta ]= \mathcal {L}[\zeta _{\iota _0}\psi _0] \end{aligned}$$

in *C* as $\delta \rightarrow 0$, where $\zeta _{\iota _0}\psi _0$ is defined by (6). Thus, there exists $\delta _0>0$ such that

$$\begin{aligned} \underline{\mathcal {L}}_{\iota _0}[\psi _0] =\underline{\mathcal {L}}[\zeta _{\iota _0}\psi _0] |_{B_{\iota _0}}=\mathcal {L}^{\delta _0}[\zeta _{\iota _0}\psi _0]|_{B_{\iota _0}} \ge \frac{1+\rho (\mathcal {L}_{\iota _0})}{2}\psi _0. \end{aligned}$$

This, together with the Gelfand’s formula and (Yi and Zhao 2023, Proposition 2.3-(i)), derives that $\rho (\underline{\mathcal {L}}_{\iota _0})\ge \frac{1+\rho (\mathcal {L}_{\iota _0})}{2}$. It then follows from the definition of $\rho _{\underline{\mathcal {L}}}$ that $\rho _{\underline{\mathcal {L}}}>1$. $\square$

### Theorem 1

Let **(F)**, **(G)**, and **(A)** hold. Then the following statements are valid:

(i) If $\rho _{\mathcal {L}}>1$, then *Q* has a nontrivial fixed point *W* such that $\lim \limits _{\Vert x\Vert \rightarrow \infty }W(x)=0$. If addition, $f(x_0,\cdot )$ is strictly subhomogeneous for some $x_0\in \mathbb {R}^N$, then
  $$\begin{aligned} \lim \limits _{n\rightarrow \infty }||Q^n[\varphi ]-W||_{L^\infty (\mathbb {R}^N,\mathbb {R})}=0, \quad \forall \varphi \in C_+\backslash \{0\}. \end{aligned}$$
(ii) If $0<\rho _{\mathcal {L}}<1$, then for any $\varphi \in C_+$, we have
  $$\begin{aligned} \lim \limits _{n\rightarrow \infty }||Q^n[\varphi ]||_{L^\infty (\mathbb {R}^N,\mathbb {R})}=0, \end{aligned}$$
  and hence, *Q* has no nontrivial fixed point *W* in $C_+$.

### Proof

(i) By Lemma 6, we see that there exists $\delta _0>0$ such that $\rho _{\underline{\mathcal {L}}}>1$, where $\underline{\mathcal {L}}=\mathcal {L}[\cdot ; \Lambda _{\eta , \delta _0,\frac{1}{\delta _0}},\Gamma _{\theta , \delta _0,\frac{1}{\delta _0}}]$. It follows from the definitions of $\Lambda$ and $\Gamma$ that $\lim \limits _{\Vert x\Vert \rightarrow \infty }\Lambda _{\eta , \delta _0,\frac{1}{\delta _0}}(x)=0$ and $\lim \limits _{\Vert x\Vert \rightarrow \infty }\Gamma _{\theta , \delta _0,\frac{1}{\delta _0}}(x)=-\mu ^*$. Applying $\underline{\mathcal {L}}$ to Lemma 3, we find that $\underline{\mathcal {L}}$ satisfies all the assumptions of (Yi and Zhao 2023, Corollary 2.7).

For any $$\varphi \in C^\circ _+$$, there exists $$r>r^{**}$$ such that $$\varphi \in C_{r^{**}}$$. In light of Lemma 4 and 5, we conclude that there exists $$\xi ^{*}:=\xi ^{*}_{\delta _0}\in (0, r)$$ such that

$$\begin{aligned} f(x,u)\ge \Gamma _{\theta ,\delta _0,\frac{1}{\delta _0}}(x)u,\, \, \quad (x,u)\in \mathbb {R}^{N} \times [0,\xi ^{*}], \end{aligned}$$

and

$$\begin{aligned} g(x,u)\ge \Lambda _{\eta ,\delta _0,\frac{1}{\delta _0}}(x)u,\, \, \quad (x,u)\in \mathbb {R}^{N} \times [0,\xi ^{*}]. \end{aligned}$$

Take $$\xi ^{**}\in (0,\xi ^{*}]$$ such that $$\Phi [1,\xi ^{**};\Gamma _{\theta ,\delta _0,\frac{1}{\delta _0}}]\le \xi ^{*}$$. By the comparison principle, it follows that

$$\begin{aligned} r\ge Q[\varphi ]\ge \underline{\mathcal {L}}[\psi ],\, \, \, (\varphi ,\psi )\in C_{r}\times C_{\xi ^*}\, \, \text { with } \, \, \psi \le \varphi . \end{aligned}$$

This, combined with proposition 3-(i), verifies all the assumptions in (Yi and Zhao 2023, Theorem 3.5 (i)). Therefore, the first statement of (i) holds.

According to (Yi and Zhao 2023, Theorem 3.5 (i)), we only need to prove that *Q* has the unique nontrivial fixed point $\widetilde{W}\in C_{r}$. Otherwise, suppose that there exists $\widetilde{W}_1\in C_{r}$ such that $Q[\widetilde{W}_1]=\widetilde{W}_1$ and $\widetilde{W}_1\ne \widetilde{W}$. It follows from Proposition 2 and Proposition 3-(i) that $\widetilde{W}, \widetilde{W}_1\in C_{r} \cap C^\circ _+$. Let

$$ \mathcal {D}=\{\psi \in C_{r}: \psi \ge \widetilde{W} \text{ and } \psi \ge \widetilde{W}_1\}. $$

Then $\mathcal {D}$ is a convex, closed, and nonempty subset of *C* such that $\mathcal {D}\subseteq C_{r}$ and $Q[\mathcal {D}]\subseteq \mathcal {D}$. This, combined with the compactness of $Q[\cdot ]$ and the Schauder’s fixed point theorem, shows that there exists $\widetilde{W}_+\in \mathcal {D}$ such that $Q[\widetilde{W}_+]=\widetilde{W}_+$, and thus $\widetilde{W}_+\ge \widetilde{W}$ and $\widetilde{W}_+\ne \widetilde{W}$. Therefore, $W_+:=\Phi _\tau [\widetilde{W}_+]>W:=\Phi _\tau [\widetilde{W}]$, where $W_+$ and *W* are positive fixed points of $\Phi _\tau \circ G$.

By **(A)**, we see that there exists $\varepsilon _0>0$ such that

$$\begin{aligned} (\limsup \limits _{\Vert x\Vert \rightarrow \infty }\eta (x)+\varepsilon _0) e^{(\limsup \limits _{\Vert x\Vert \rightarrow \infty }\theta (x)+\varepsilon _0)\tau } <1. \end{aligned}$$

By **(F)** and **(G)**, we see that there exists $\rho _0>0$ such that for any $(x,u)\in [\rho _0,\infty )\times [0, r]$, we have

8 $$\begin{aligned} f(x,u)\le \xi (x) u \le f_{\varepsilon _0}u:= ( \limsup \limits _{\Vert x\Vert \rightarrow \infty } \theta (x)+\varepsilon _0)u,\, \end{aligned}$$

and

9 $$\begin{aligned} g(x,u)\le \eta (x) u \le g_{\varepsilon _0}u:= ( \limsup \limits _{\Vert x\Vert \rightarrow \infty } \eta (x)+\varepsilon _0)u. \end{aligned}$$

Let

$$ a^* = \sup \left\{ a\in \mathbb {R}_+: \begin{array}{l} \Phi _t\circ G[W(\cdot )](x)\ge \Phi _t\circ G[aW_+(\cdot )](x) \text{ and } \\ W(x)\ge aW_+(x) \text{ for } (t,x)\in [0,\tau ]\times B_{\rho _0}. \end{array} \right\} $$

Clearly, $0\le a^*\le 1$ due to $W_+\ge W$. We divide the remaining proof into three steps.

**Step 1.** Prove that $0<a^*\le 1$.

Pick $a_1=\frac{\min \limits _{x\in B_{\rho _0}}W(x)}{\max \limits _{x\in B_{\rho _0}}W_+(x)}$. Then $W(x)\ge a_1W_+(x)$ for all $x \in B_{\rho _0}$. Let $q_0=\min \{ \Phi _t\circ G[W(\cdot )](x):(t,x) \in [0,\tau ]\times B_{\rho _0}\}$. Then from the monotonicity of $\Phi _t\circ G$ and the continuity of $\Phi _t\circ G[N](x)$ with respect to the continuity of (*t*, *x*, *N*), we see that there exist $N_0\in (0,re^{|K|\tau })$ and $a_2=\frac{N_0}{re^{|K|\tau }}$ such that

$$\begin{aligned} q_0\ge \Phi _t\circ G[N_0](x)=\Phi _t\circ G[a_2 re^{|K|\tau }](x)\ge \Phi _t\circ G[a_2 W_+(\cdot )](x), \end{aligned}$$

and hence $\Phi _t\circ G[W(\cdot )](x)\ge \Phi _t\circ G[a_2 W_+(\cdot )](x)$ for all $(t,x) \in [0,\tau ]\times B_{\rho _0}$. Thus, we conclude that $a^*\ge \min \{a_1,a_2\}>0$.

**Step 2.** Prove that $W\ge a^* W_+$.

By induction, we first establish the following iterative inequality:

10 $$\begin{aligned} W(x)-a^*W_+(x)+\Lambda [g_{\varepsilon _0} e^{f_{\varepsilon _0} \tau }]^n\ge 0 \text{ for } n\in \mathbb {N}, x\in \mathbb {R}^N, \end{aligned}$$

where $$\Lambda =1+\sup \limits _{x\in \mathbb {R}^N}|W(x)|+\sup \limits _{x\in \mathbb {R}^N}|W_+(x)|$$.

Due to the choice of $\Lambda$, (10) holds when $n=0$. Suppose that (10) holds for $n=m$, that is, $W(x)-a^*W_+(x)+\Lambda [g_{\varepsilon _0} e^{f_{\varepsilon _0} \tau }]^{m}\ge 0$ for $x\in \mathbb {R}^N$. We now show that (10) also holds for $n=m+1$. Define $v:[0,\tau ]\times \mathbb {R}^N\rightarrow \mathbb {R}$ by

$$\begin{aligned} v(t,x)=\Phi _t\circ G[W(\cdot )](x)-\Phi _t\circ G[a^*W_+(\cdot )](x)+\Lambda g_{\varepsilon _0} e^{f_{\varepsilon _0} t}[g_{\varepsilon _0} e^{f_{\varepsilon _0} \tau } ]^{m} \end{aligned}$$

for $(t,x)\in [0,\tau ]\times \mathbb {R}^N$. By the definitions of $a^*$ and *v*, we see that for any $(t,x)\in [0,\tau ]\times B_{\rho _0}$, we have $v(t,x)\ge 0$. By induction and (9), for any $x\notin B_{\rho _0}$, we know that

$$\begin{aligned} v(0,x)= &  G[W](x)-G[a^*W_+] (x)+\Lambda g_{\varepsilon _0} [g_{\varepsilon _0} e^{f_{\varepsilon _0} \tau } ]^{m} \\= &  \int _{\mathbb {R}^N} \mathcal {K}(x-y)[g(y,W(y))-g(y,a^*W_+(y))]\textrm{d}y+\Lambda g_{\varepsilon _0}[g_{\varepsilon _0} e^{f_{\varepsilon _0} \tau } ]^{m} \\\ge &  \int _{y\in \mathbb {R}^N \backslash B_{\rho _0}} \mathcal {K}(x-y)[g(y,W(y))-g(y,a^*W_+(y))]\textrm{d}y +\Lambda g_{\varepsilon _0}[g_{\varepsilon _0} e^{f_{\varepsilon _0} \tau } ]^{m} \\= &  \int _{y\in \mathbb {R}^N \backslash B_{\rho _0}} \mathcal {K}(x-y)h(y)[W(y)-a^*W_+(y)]\textrm{d}y +\Lambda g_{\varepsilon _0}[g_{\varepsilon _0} e^{f_{\varepsilon _0} \tau } ]^{m} \\\ge &  -\int _{y\in \mathbb {R}^N \backslash B_{\rho _0}} \mathcal {K}(x-y)g_{\varepsilon _0} \Lambda [g_{\varepsilon _0} e^{f_{\varepsilon _0} \tau } ]^{n_0}\textrm{d}y +\Lambda g_{\varepsilon _0}[g_{\varepsilon _0} e^{f_{\varepsilon _0} \tau } ]^{m} \\\ge &  0, \end{aligned}$$

where $h(y)=\int _0^1\frac{\partial g}{\partial u}(y,sW(y)+(1-s)a^*W_+(y))\textrm{d}s$. These, combined with the definition of $\Phi _t$ and (8), verify that *v* satisfies

$$\begin{aligned} \left\{ \begin{array}{ll} \frac{\partial v}{\partial t} (t,x) \ge \nabla \cdot (D\nabla v(t,x)-q v (t,x))+l(t,x)v(t,x), & (t,x) \in [0,\tau ]\times \mathbb {R}^N \backslash B_{\rho _0}, \\ v(0,x)\ge 0, & x\notin B_{\rho _0}, \\ v(t,x)\ge 0, & (t,x)\in [0,\tau ]\times B_{\rho _0}, \end{array} \right. \end{aligned}$$

where $l(t,x)=\int _0^1\frac{\partial f}{\partial u}(x,s\Phi _t\circ G[W(\cdot )](x)+(1-s)\Phi _t\circ G[a^*W_+(\cdot )](x))\textrm{d}s$. This gives rise to $v(t,x)\ge 0$ for all $(t,x)\in [0,\tau ] \times \mathbb {R}^N$ due to Phragmén-Lindelöf maximum principle. By the definition of *v*, we see that $v(\tau ,x)= W(x)-a^*W_+(x)+\Lambda [g_{\varepsilon _0} e^{f_{\varepsilon _0} \tau }]^{m+1}\ge 0 \text{ for } x\in \mathbb {R}^N$ due to $W_+$ and *W* are positive fixed points of $\Phi _\tau \circ G$. By induction, we have proved (10). Letting $n\rightarrow \infty$ in (10) produces $W\ge a^*W_+$.

**Step 3.** Prove that $a^*=1$.

By way of contradiction, we assume that $a^*<1$. Select $\mu =1+\sup \Big \{|\frac{\partial f}{\partial u}(x,u)|:(x,u)\in \mathbb {R}^N\times [0,re^{|K\tau |}]\Big \}$. By the definition of $a^*$ and the conditions of *f*, *g*, we see that, for any $x\in \mathbb {R}^N$,

$$\begin{aligned} W(x)= &  S(\tau )[G[W]](x) +\int _0^\tau S(\tau -s)\Big [\mu \Phi _s\circ G[W](\cdot ) \\ &  + f(\cdot ,\Phi _s\circ G[W](\cdot )) \Big ](x)\textrm{d} s \\\ge &  S(\tau )[G[a^*W_+(\cdot )]](x) + \int _0^\tau S(\tau -s)\Big [\mu \Phi _s\circ G[a^*W_+(\cdot )] \\ &  + f(\cdot ,\Phi _s\circ G[a^*W_+(\cdot )]) \Big ](x)\textrm{d} s \\> &  a^* S(\tau )[G[W_+]](x)+ a^*\int _0^\tau S(\tau -s)\Big [\mu \Phi _s\circ G[W_+](\cdot ) \\ &  + f(\cdot ,\Phi _s\circ G[W_+](\cdot )) \Big ](x)\textrm{d} s \\= &  a^*W_+(x), \end{aligned}$$

where, for any $\phi \in C$,

$$ S(t)[\phi ](x)= {\left\{ \begin{array}{ll} \phi (x), & t=0, \\ \frac{e^{-\mu t}}{\sqrt{|D|}(4 \pi t)^{\frac{1}{2}}} \int _{\mathbb {R}^{N}} e^{-\frac{(x-q t-y)^{T} D(x-q t-y)}{4 t}}\phi (y)\textrm{d}y, \qquad & t>0. \end{array}\right. } $$

Thus, there exists $\epsilon >0$ such that

$$\begin{aligned} W(x)-a^*W_+(x)\ge \epsilon \ge \epsilon \Big [\frac{a^*W_+(x)}{1+\sup \limits _{x\in B_{\rho _0}}a^*W_+(x)}\Big ] \end{aligned}$$

for $x\in B_{\rho _0}$. Take $\delta =\frac{a^*\epsilon }{1+a^*\sup \limits _{x\in B_{\rho _0}}W_+(x)}$. As a result, we find $W(x)\ge (a^*+\delta )W_+(x)$ for all $x\in B_{\rho _0}$, which contradicts the definition of $a^*$. Therefore, $a^*=1$. This gives rise to $W=W_+$, and hence $\widetilde{W}=\widetilde{W}_+$. This completes the proof.

(ii) By $0<\rho _{\mathcal {L}}<1$ and Lemma 3, we may use Yi and Zhao 2023, Theorem 2.5-(ii) to derive the existence of $d_0>0$, $\sigma ^*>0$, $\rho ^*\in (0,1)$ and $\eta _\infty ^*\in C_+^\circ$ such that

$$\begin{aligned} |\eta _\infty ^*|_\infty =1, \quad \widehat{\mathcal {L}}_{\sigma ^*,1+d_0}[ \eta _\infty ^*]=\rho ^*\eta _\infty ^* < \eta _\infty ^*. \end{aligned}$$

By slightly modifying Yi and Zhao 2023, Theorem 3.5-(ii),Theorem 3.2, we only need to prove that for any $\varphi \in C_+$, there exists $n\in \mathbb {N}$ such that

$$\begin{aligned}\omega (\varphi )\subseteq C_{n \eta _\infty ^*}. \end{aligned}$$

It follows from Lemma 3-(ii) that there exists $\delta _0\in (0,1), s_0\in (0,\infty )$ and $n_0\in \mathbb {N}$ such that

$$\begin{aligned} \mathcal {L}^{n_0}[1](x)< \delta _0, \qquad \Vert x\Vert \ge s_0. \end{aligned}$$

Let $$b^*=\inf \{b\in \mathbb {R}_+:\psi (x)\le b \eta _{\infty }^*(x), \quad (x,\psi )\in B_{s_0}\times \omega (\varphi )\}$$. Then $$b^*\in \mathbb {R}_+$$ and $$\omega (\varphi )|_{B_{s_0}}\le b^* \eta _{\infty }^*|_{B_{s_0}}$$. We claim that $$\omega (\varphi )\le b^* \eta _{\infty }^*$$. Otherwise, we have $$\gamma :=\sup \{h(x,\psi ):(x,\psi )\in \mathbb {R}^N\times \omega (\varphi )\}>0$$, where $$h(x,\psi ):=\psi (x)-b^*\eta _{\infty }^*(x)$$. By Proposition 4, we see that $$\limsup \limits _{\Vert x\Vert \rightarrow \infty }\{\sup \psi (x):\psi \in \omega (\varphi )\}=0$$. Then, there exists $$\alpha _0>s_0$$ such that $$h(x,\psi )<\frac{\gamma }{2}$$ for $$\Vert x\Vert >\alpha _0$$ and $$\psi \in \omega (\varphi )$$. Thus, we have $$\gamma =\sup \{h(x,\psi ):(x,\psi )\in B_{\alpha _0}\times \omega (\varphi )\}$$ due to the definition of $$\gamma $$. This, combined with the invariance of $$\omega (\varphi )$$, implies that there exists $$(x^*,\psi ^*,\psi ^{**})\in B_{\alpha _0}\backslash B_{s_0}\times \omega (\varphi )\times \omega (\varphi )$$ such that

11 $$\begin{aligned} \psi ^*(x^*)-b^*\eta _{\infty }^*(x^*)=\sup \left\{ \psi (x)-b^*\eta _{\infty }^*(x): (x,\psi )\in \mathbb {R}^{N}\times \omega (\varphi )\right\} >0 \end{aligned}$$

and $$\psi ^*=Q^{n_0}[\psi ^{**}]$$.

By the choices of $$b^*,x^*,\rho ^*,\psi ^*,\psi ^{**},s_0$$ and (Yi and Zhao 2023, Lemma 2.1-(i)), we see that

$$\begin{aligned} &  \psi ^*(x^*)-b^*\eta _{\infty }^*(x^*) \\= &  Q^{n_0}[\psi ^{**}](x^*) -b^* \eta _{\infty }^*(x^*) \\\le &  \mathcal {L}^{n_0}[\psi ^{**}](x^*) -\frac{b^*}{(\rho ^*)^{n_0}} ( \widehat{\mathcal {L}}_{\sigma ^*,1+d_0})^{n_0}[ \eta _\infty ^*](x^*) \\\le &  \mathcal {L}^{n_0}[\psi ^{**}](x^*) -\frac{b^*}{(\rho ^*)^{n_0}} \mathcal {L}^{n_0}[ \eta _\infty ^*](x^*) \\\le &  \mathcal {L}^{n_0}[\psi ^{**}-b^*\eta _\infty ^*](x^*) \\\le &  \sup \{\psi ^{**}(x)-b^*\eta _\infty ^*(x):x\in \mathbb {R}^N \} \mathcal {L}^{n_0}[1](x^*). \end{aligned}$$

This, together with (11), yields the following,

$$\begin{aligned} \psi ^*(x^*)-b^*\eta _{\infty }^*(x^*)\le &  (\psi ^*(x^*)-b^*\eta _{\infty }^*(x^*))\times \mathcal {L}^{n_0}[1](x^*) \\\le &  \delta _0(\psi ^*(x^*)-b^*\eta _{\infty }^*(x^*)), \end{aligned}$$

a contradiction. Consequently, $\omega (\varphi )\le b^* \eta _{\infty }^*$, and hence, statement (ii) holds. $\square$

## Numerical simulations

In this section, we perform numerical investigations to examine the impact of key parameters on population dispersal and extinction, as well as to validate our theoretical findings. The simulations are designed to illustrate the model’s behavior and highlight the essential aspects of the theoretical results.

Following conditions **(F)** and **(G)**, for simplicity and without loss of generosity, we investigate the population dynamics of a single population by setting $N=1$. We also choose $\tau =1, q>0$, and the mortality and reproduction functions are taken to be of the form $f(x,u)=-\alpha (x)u-u^2$ and $g(x,u)={\frac{r(x)u}{1+u}}$, respectively, where

$$\begin{aligned} r(x)= \left\{ \begin{array}{ll} 1, & x\in (-\infty ,-2l], \\ \frac{x}{l}+ 3, & x\in (-2l,-l), \\ 2, & x\in [-l,l], \\ -\frac{x}{l}+ 3, & x\in (l,2l), \\ 1, & x\in [2l,\infty ), \end{array} \right. \qquad \alpha (x)= \left\{ \begin{array}{ll} 1, & x\in (-\infty , -2m], \\ -\frac{x}{m}-1, & x\in (-2m,-m), \\ 0, & x\in [-m,m], \\ \frac{x}{m}-1, & x\in (m,2m), \\ 1, & x\in [2m,\infty ). \end{array} \right. \end{aligned}$$

Here, *m* and *l* are selected positive constants that measure the sizes of favorable habitat regions. It is easy to see that the Assumption **(A)** holds. Taking $K=0,L=2$, one can verify that *f* and *g* satisfy all the conditions in the Assumptions **(F)** and **(G)**. In this setting, we assume that the preferred habitat is centralized. Specifically, within the region $[-m,\,m]$, environmental conditions are optimal for the species, ensuring that there is no location-dependent mortality. Outside the core region, habitat quality declines linearly until it reaches the boundaries at $-2m$ and 2*m*, beyond which unfavorable conditions remain constant and extend indefinitely. Similarly, the spatial distribution of the suitability for breeding for the next generation is characterized by the parameter *l*. The values of *m* and *l* determine the specific combinations of favorable habitat conditions for survival and/or reproduction in the central portion of the one-dimensional spatial domain. As individuals disperse outward along the $x-$axis, environmental conditions gradually deteriorate, leaving only one favorable condition and eventually becoming completely unsuitable for both survival and reproduction. Additionally, we adopt the Gaussian distribution as the kernel function governs adult dispersal for reproduction, reflecting localized movement centered around breeding areas.

In addition, to show the alternative pattern of favorable and unfavorable habitat in terms of reproduction, we also introduce $r_1(x)$ in a periodic manner in our numerical analysis.

$$\begin{aligned} r_1(x)= \left\{ \begin{array}{ll} 2, & x\in ((5k-1)l,(5k+1)l], \\ -\frac{x-5kl}{l}+3, & x\in ((5k+1)l,(5k+2)l], \\ 1, & x\in ((5k+2)l,(5k+3)l], \\ \frac{x-5kl}{l}+3, & x\in ((5k+3)l,(5k+4)l], k=0,\pm 1,\pm 2,.... \end{array} \right. \end{aligned}$$

Now, we begin by presenting numerical simulations to show how the behavior of the solution to equation (2) depends on the choice of initial conditions in the positive cone $C_+$. Without loss of generosity, we consider three different initial distributions,

$$\begin{aligned} u_1(x)=1, x\in \mathbb {R}, \quad u_2(x)=\left\{ \begin{aligned}&1, x\in [-10, \ 10],\\ &0, otherwise\end{aligned}\right. , \quad u_3(x)=\left\{ \begin{aligned}&1, x\in [-9, \ -7]\cup [7, \ 9],\\ &0, otherwise.\end{aligned}\right. \end{aligned}$$

**Fig. 1:**
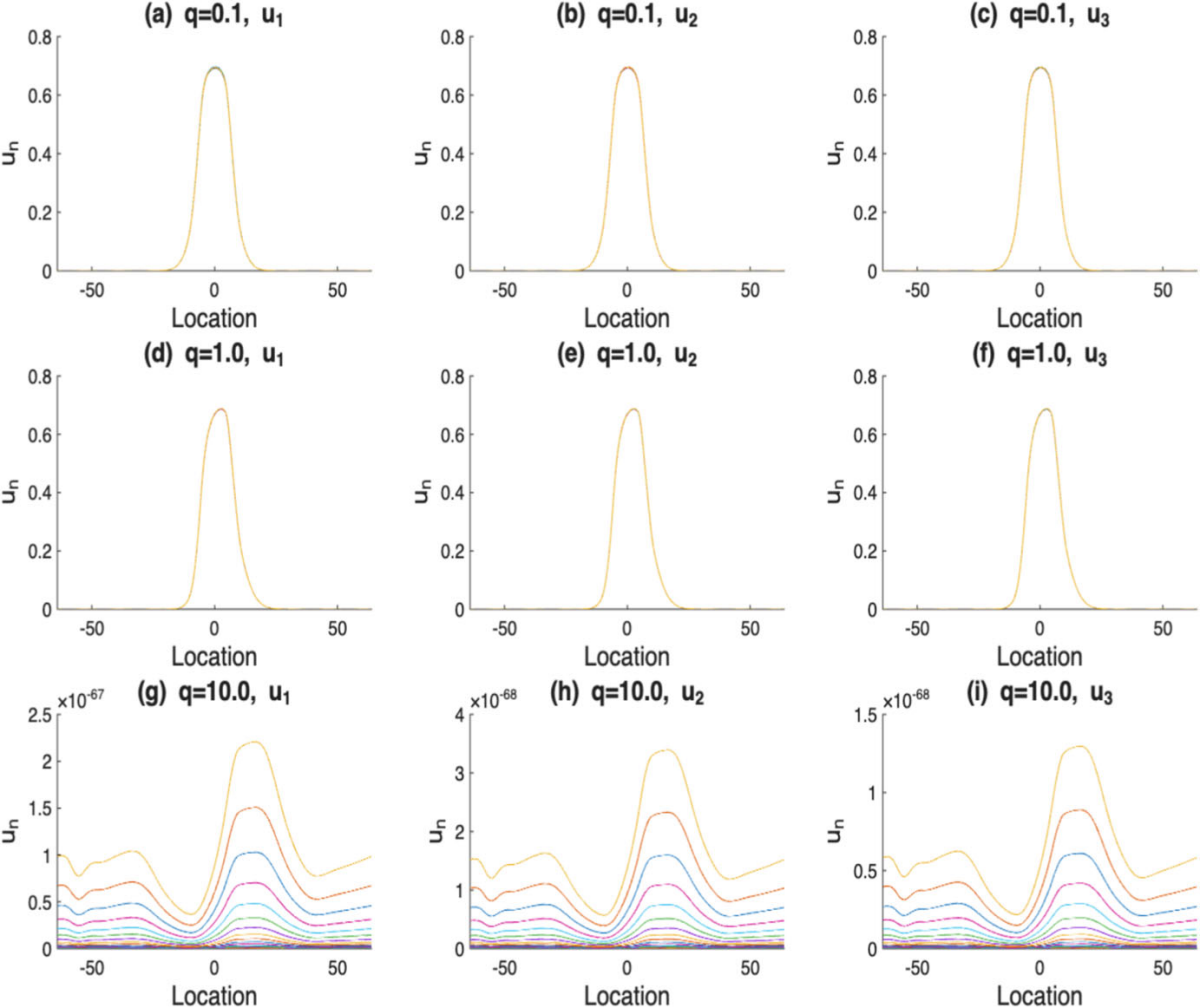
The solutions $u_n$, where $n=400,..,500$. Here, we have $\alpha (x)$ with $m=5$, *r*(*x*) with $l=10$, $\mathcal {K}(x)=\frac{1}{2\sqrt{2\pi }}e^{-\frac{x^2}{8}}$ and *q* varies from $0.1, 1 \text {\ to\ } 10$. Subplots in each column share the same initial condition

As shown in Fig. 1 (a)-(c), the solutions either converge to the same positive steady state or decay to zero, regardless of the initial distribution. Furthermore, the simulations in Fig. 1 (a) (d) (g) demonstrate that a higher drift rate *q* accelerates extinction faster, where *q* increases from 0.1, 1 to 10, respectively, and the solution degenerates to zero more rapidly with larger *q*.

**Fig. 2:**
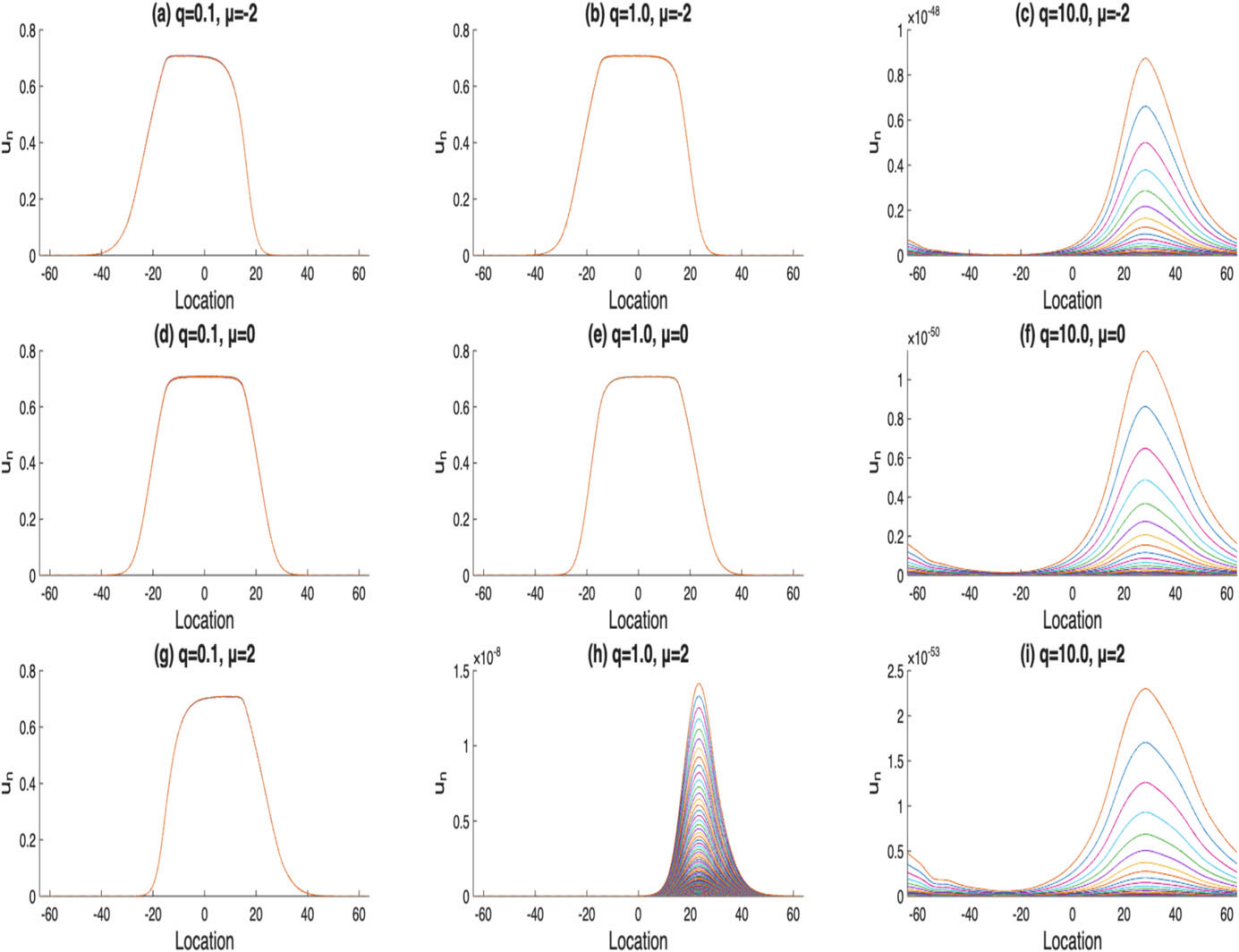
The solutions $u_n$, where $n=400,..,500$. Here, $\alpha (x)$ with $m=5$, $r_1(x)$ with $l=10$, $\mathcal {K}(x)=\frac{1}{2\sqrt{2\pi }}e^{-\frac{(x-\mu )^2}{8}}$, *q* varies from 0.1, 1 to 10, and $\mu$ varies from $-2, 0$ to 2

**Fig. 3:**
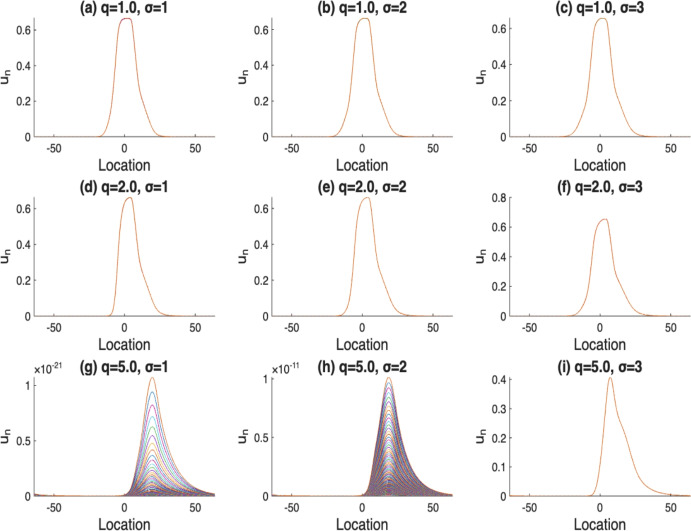
The solutions $u_n$, where $n=400,..,500$. Here, $\alpha (x)$ with $m=5$, *r*(*x*) with $l=10$, $\mathcal {K}(x)=\frac{1}{\sigma \sqrt{2\pi }}e^{-\frac{x^2}{2\sigma ^2}}$, *q* varies from 1, 2 to 5, and $\sigma$ varies from 1, 2 to 3

In the following investigations, we adopt $u_1$ as the initial condition for all subsequent simulations. We now examine the influence of the Gaussian dispersal kernels on the dynamics of the model solutions. Our results show that when the mean value for the Gaussian distribution shifts towards the direction of population movement, the likelihood of population extinction increases, as illustrated in the second column of Fig. 2 (b), (e), and (h). In contrast, when varying the variance of the Gaussian kernel, we observe that a larger variance can promote the persistence of population easier – even under high diffusion rates. This behavior is illustrated in Fig. 3 (i), where the population remains persistent despite strong diffusion, due to the spread over the dispersal kernel.

**Fig. 4:**
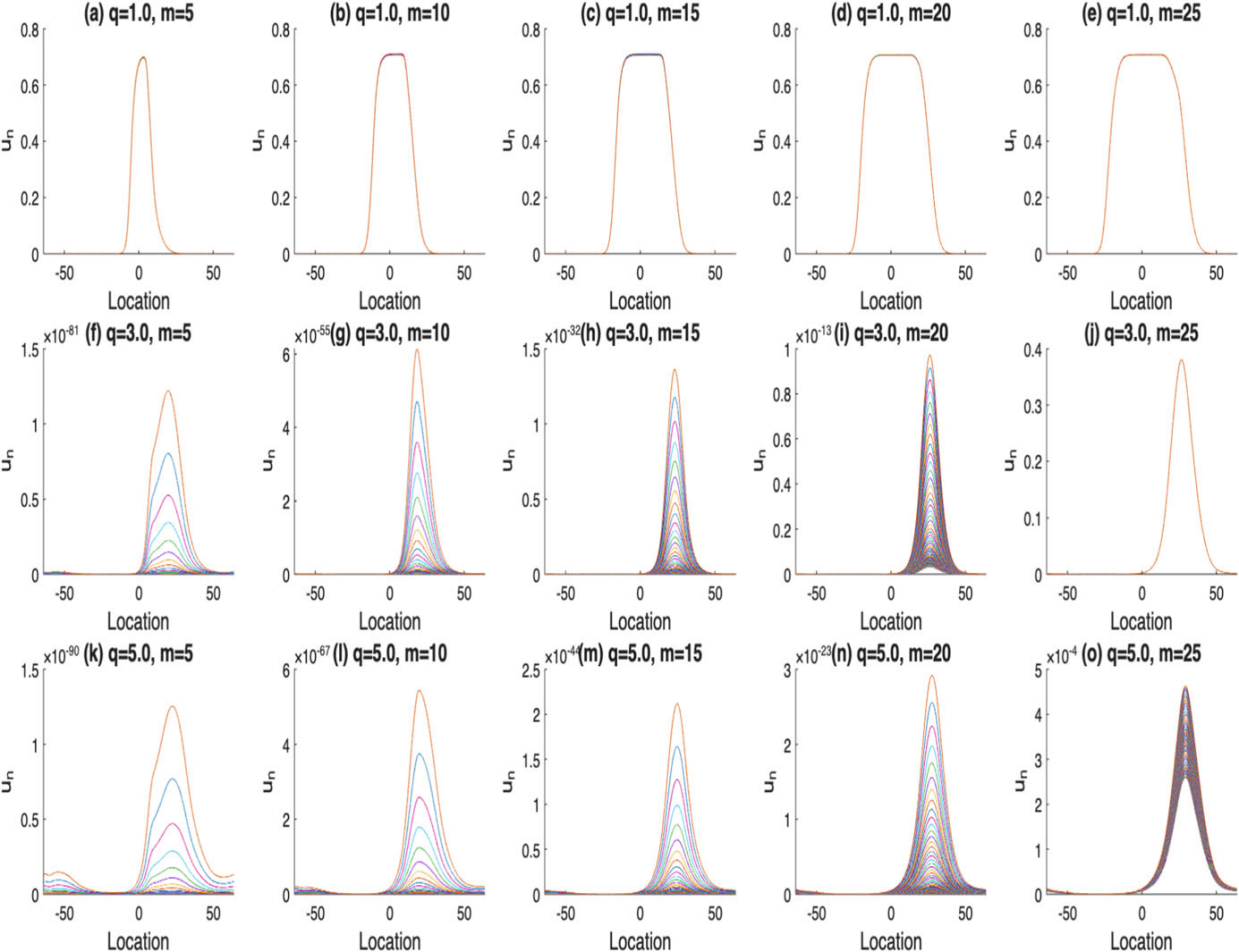
The solutions $u_n$, where $n=400,..,500$. Here, *m* varies from $5,10, 15, 20 \text { to } 25$ in $\alpha (x)$, $r_1(x)$ with $l=15$, and $\mathcal {K}(x)=\frac{1}{2\sqrt{2\pi }}e^{-\frac{x^2}{8}}$

Next, we investigate the impact of the parameters *m* and *l*, which characterize spatial heterogeneity and the extent of favorable conditions for survival and growth, respectively, on the likelihood of population persistence. Increasing these two parameters expands the safe zones within a habitat where the population can thrive, thereby enhancing the persistence of the population. Specifically, a larger *m* widens the area of favorable habitat, mitigating the adverse effect of rapid diffusion that can lead to population decline or extinction. This relation is evident in Fig. 4, where higher diffusion rates (vertical comparison) generally decrease persistence probability, but larger *m* (horizontal comparison) can counteract this trend and promote stability. Similarly, an extended region favorable to population growth (i.e., larger *l*) also promotes population survival. Although the numerical results for varying *l* closely resemble those in Fig. 4, they are not displayed here due to space constraints.

To further explore the interplay between the positive effects of the expansion of favorable habitats and the negative impacts of rapid diffusion on population persistence, we examine how these factors interact within spatially heterogeneous environments. Without loss of generality, we also choose $\alpha _1(x)$ in a periodic manner, i.e.,

$$\begin{aligned} \alpha _1(x)= \left\{ \begin{array}{ll} 0, & x\in ((5k-1)m,(5k+1)m], \\ \frac{x-5km}{m}-1, & x\in ((5k+1)m,(5k+2)m], \\ 1, & x\in ((5k+2)m,(5k+3)m], \\ -\frac{x-5km}{m}-1, & x\in ((5k+3)m,(5k+4)m], k=0,\pm 1,\pm 2,.... \end{array} \right. \end{aligned}$$

**Fig. 5:**
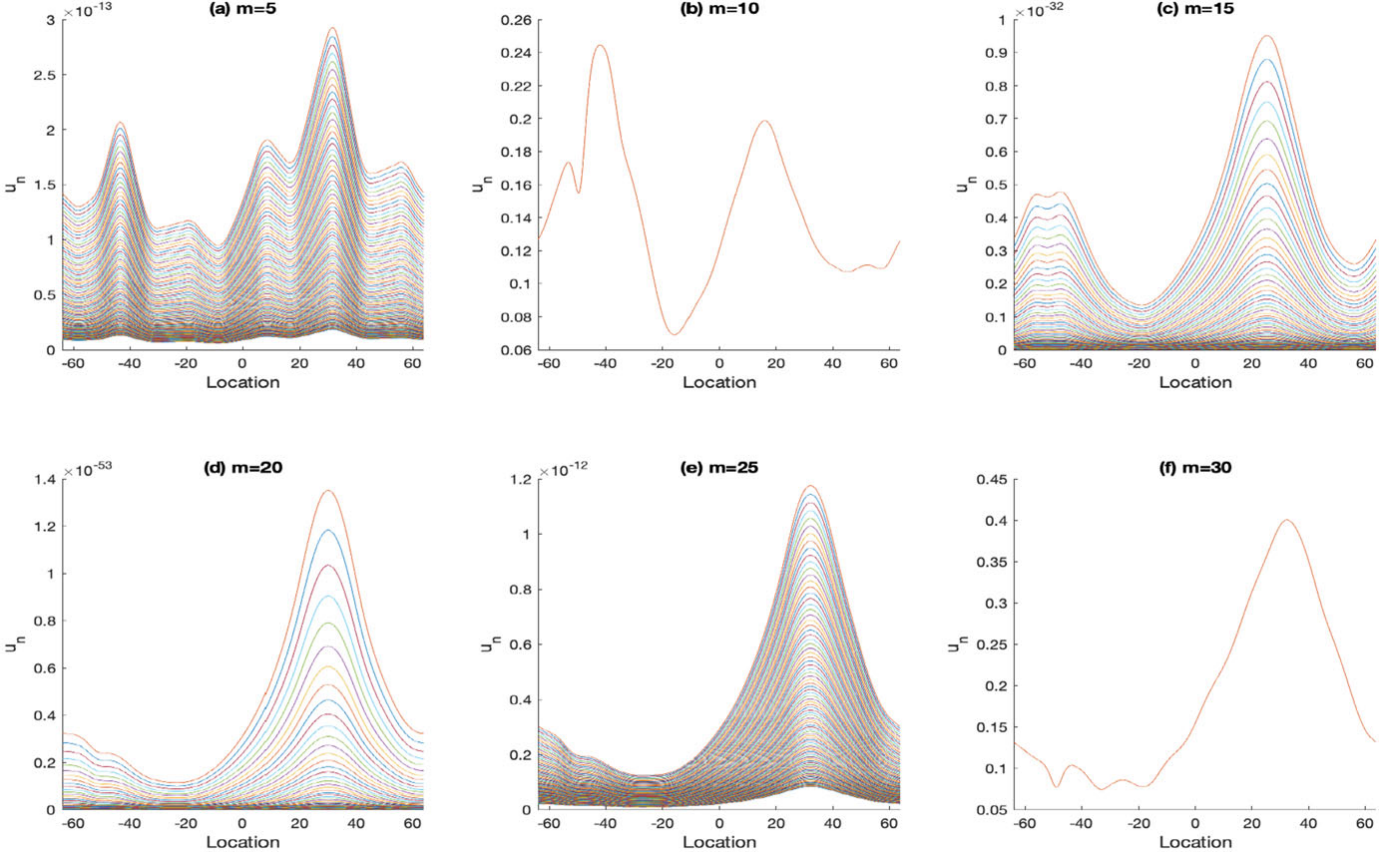
The solutions $u_n$, where $n=900,..,1000$. Here, *m* varies from $10, 15, 18, 20, 25 \text { to } 30$ in $\alpha _1(x)$ and $r_1(x)$ with $l=15$, $q=15$, and $\mathcal {K}(x)=\frac{1}{2\sqrt{\pi }}e^{-x^2}$

The observed dynamics indicate that when $m=10$ or 15 and $l=15$, the population reaches a positive steady state, benefiting from periodically emerging favorable regions for both survival and reproduction, as illustrated in Fig. 5 (a) and (b). However, increasing *m* to 18 and 20, while keeping *l* constant, leads population decline and eventual extinction, as presented in Fig. 5 (c) and (d). Interestingly, further increasing *m* to 25 and 30 results in the population stabilizing once again at a positive steady state, as shown in Fig. 5 (e) and (f). This pattern suggests an alternating sequence of persistence and extinction as *m* increases from 10 to 30.

Note that the theoretical analysis is carried out in the general setting $\mathbb {R}^N$, all numerical simulations are performed in the one-dimensional space $\mathbb {R}$. This restriction is mainly for simplicity of presentation and computational convenience. The one-dimensional case already captures the essential dynamical features predicted by the theory and allows for a clearer visualization of the solution profiles.

## Discussion

In this paper, we study a two stage-structured population model in a heterogeneous environment, based on the work of Vasilyeva et al. (2016). Our model describes insects/aquatic species inhabiting streams, characterized by two distinct, non-overlapping life-cycle stages, i.e., an aquatic larval stage and a winged adult stage. Spatial heterogeneity is introduced through a stage-specific larval mortality function of the larval stage and a birth function that governs egg deposition and survival to the adult stage. The model is formulated using an impulsive reaction-advection-diffusion equation with non-local impulse. Extending the results of Vasilyeva et al. (2016) from homogeneous environments to spatially heterogeneous environments, we establish the threshold dynamics in spatially heterogeneous settings. By utilizing the theory of the asymptotic spectral radius of operators and analyzing key qualitative properties of the associated discrete map, we apply the abstract theory in non-translation-invariant spaces to derive sufficient conditions of threshold dynamics.

**Fig. 6:**
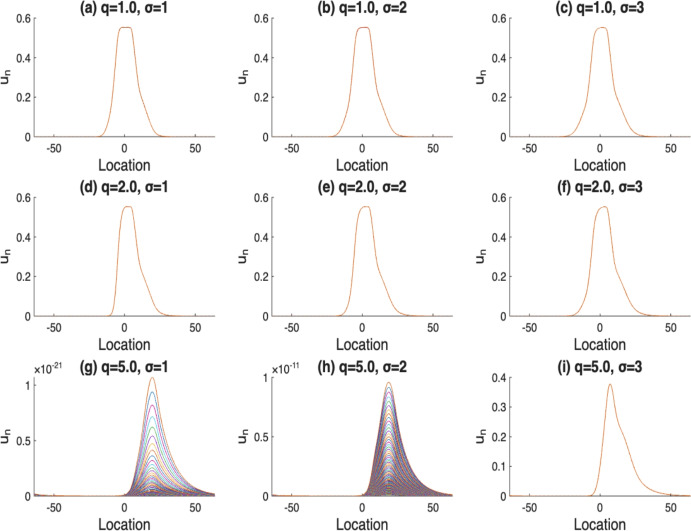
The solutions $u_n$, where $n=400,..,500$. Here, $\alpha (x)$ with $m=5$, *r*(*x*) with $l=10$, $\mathcal {K}(x)=\frac{1}{\sigma \sqrt{2\pi }}e^{-\frac{x^2}{2\sigma ^2}}$, *q* varies from 1, 2 and 5, $\sigma$ varies from 1, 2 and 3, and $g(x)=r(x)u(1-u)$

The numerical simulations in Section 4 focus on the case where *g* is monotone. However, similar dynamical behaviors can also be observed for some non-monotone functions *g*. For instance, when $g(x)=r(x)u(1-u)$ and $u_0\in [0,1]$, it is easy to verify that $u_n\in [0,1]$ for all $n\ge 0$. The corresponding numerical results are shown in Fig. 6. Figure 6 is very similar to Fig. 3, with only slight differences in population density. This observation leads us to conjecture that for some non-monotonic functions *g*, a similar threshold dynamics result may still hold. We will leave this investigation for future work.

We find that population persistence is not influenced by the initial spatial distribution, but is strongly affected by the size of favorable habitats, the drift rate of the population, and the characteristics of the dispersal kernel. These findings are consistent with previous results in spatial ecological studies (e.g., (Alqawasmeh and Lutscher 2019; Fahrig and Nuttle 2005; Lutscher et al. 2006)), and underscore the critical role of spatial heterogeneity in shaping population dynamics. In addition, we show that the size and spatial arrangement of favorable habitats significantly influence population stability and persistence. In particular, suitable habitat configurations can mitigate the negative effects of rapid diffusion driven by stream flow or other external forces. However, the interaction between habitat structure and diffusion is complex: while expanding favorable regions generally promotes population growth, excessive diffusion can offset these benefits by dispersing individuals too thinly across the landscape.

In this study, we also explore scenarios where favorable habitats are arranged periodically, i.e., distributed in a regular, repeating spatial pattern. Such spatial structuring can enhance population persistence by providing consistent support against the destabilizing effects of diffusion, particularly in heterogeneous environments. Our numerical results suggest that aligning the spatial scale of habitat patches with the diffusion characteristics of the population is crucial for maintaining population stability. Furthermore, the findings indicate the potential existence of an optimal trade-off between habitat favorability and drift rate that maximizes long-term population persistence.

In Fig 4, we present results using *m* as a bifurcation parameter to illustrate the transition between persistence and extinction. A similar numerical behavior is also observed for the parameter *l*. We conjecture that the diffusion rate *q* likewise serves as a threshold parameter governing the switch between population persistence and extinction. Specifically, an increased diffusion rate undermines the benefits brought by the expansion of favorable habitat regions. However, due to the sensitivity of model results to variations in *q*, capturing this effect numerically is more challenging. From a theoretical perspective, we aim to rigorously establish this conjecture in future work.

In this work, we began with an impulsive reaction-diffusion equation model to describe a population with two distinct life stages. Moving forward, we aim to expand our modeling framework to accommodate multi-stage structured populations, such as those with egg, larva, and adult stages to gain deeper insight into how stage-specific survival and reproduction influence overall population persistence. In addition, we plan to incorporate species interactions into our current model, particularly in the context of competition for resources for survival and reproduction. This extension will allow us to investigate the dynamics of two or more interacting species. We intend to focus on the interaction between a native species and an invasive species, examining how spatial heterogeneity and differing diffusion rates shape the outcomes of interspecies competition.

## Data Availability

This work is mainly of a theoretical and analytical nature, and hence, there is no data involved.

## Appendix

For the readers’ convenience, we recall some assumptions guided by the abstract theory in Yi and Zhao (2023).

**(AC)**: **(i) ** There exists $(\delta ^*,k^*,x^*)\in (0,1)\times \mathbb {N}\times (0,\infty )$ such that $\mathcal {L}^{k^*}[\check{1}](x)<\delta ^*\check{1}$ for all $||x||\ge x^*$. **(ii) ** For any $\sigma ,d\in (0,\infty )$, there exists $(\delta _*,k_*,x_*):=(\delta _*(\sigma ,d),k_*(\sigma ,d),$ $x_*(\sigma ,d))\in (0,1)\times \mathbb {N}\times (0,\infty )$ such that $(\widehat{\mathcal {L}}_{\sigma ,d})^{k_*}[\check{1}](x)<\delta _*\check{1}$ for all $||x||\ge x_*$.

**(AT)**: There exists linear operator $\mathcal {L}_\infty : C\rightarrow C$ such that $\lim \limits _{k,||y||\rightarrow \infty }||T_{-y}\circ \mathcal {L}^n \circ T_y[\varphi _k]-\mathcal {L}_\infty ^n[\varphi ]||=0, \, \forall n\in \mathbb {N}$ provided that $\{\varphi _k\}_{k\in \mathbb {N}\cup \{0\}}\in C_{\check{1}}$ with $\lim \limits _{k\rightarrow \infty }||\varphi _k-\varphi _0||=0$.

**(UAA)**: There exist $$r^{**}>0$$ and $$ \overline{Q},\overline{Q}^+:C_+\rightarrow C_+$$ such that there hold

(i) $$ \overline{Q}[\varphi ]\le \overline{Q}[\psi ]\le \overline{Q}[r^{**}]\le r^{**}, \, \forall \ 0\le \varphi \le \psi \in C_{ r^{**}}.$$
(ii) $$\lim \limits _{n\rightarrow \infty }||(\overline{Q}^+)^n[r^{**}](0)||=0$$, and
  $$ \liminf \limits _{||x||\rightarrow \infty } \Big [\inf \{(\overline{Q}^+)^n[r^{**}](0)-T_{-x}\circ (\overline{Q})^n \circ T_x[r^{**}](0)\}\Big ] \in \mathbb {R}_+^N, \, \forall n\in \mathbb {N}. $$

### Proof of Proposition 2

#### Proof

In view of **(F)**, we have $f(x,u)\le Ku$ for all $x\in \mathbb {R}^N$ and $u\ge \tilde{N}$ and $f(x,u)\le \partial _u f(x,0)u\le \sup \limits _{x\in \mathbb {R}^N}\partial _u f(x,0) u$ for all $(x,u)\in \mathbb {R}^N\times \mathbb {R}_+$. This, combined with $\limsup \limits _{\Vert x\Vert \rightarrow \infty }\partial _u f(x,0)<\infty$, implies that there exists $M>0$ such that

$$\begin{aligned} f(x,u)\le M, \;\; \; (x,u)\in \mathbb {R}^N\times [0,\tilde{N}]. \end{aligned}$$

Thus, there exists $\bar{f}_{K}:\mathbb {R}_+\rightarrow \mathbb {R}$ such that

$$\begin{aligned} f(x,u)\le \bar{f}_{K}(u), \;\;\; (x,u)\in \mathbb {R}^N\times \mathbb {R}_+, \end{aligned}$$

where, by the choice of *K*, we define $\bar{f}_{K}$ as follows:

$$\begin{aligned} \bar{f}_{K}(u)=\max \{Ku, M\} \; \text{ for } \; u\in \mathbb {R}_+ \end{aligned}$$

provided that $$K>0$$;

$$\begin{aligned} \bar{f}_{K}(u)=\left\{ \begin{array}{ll} M, & u\in [0,\tilde{N}], \\ -M(u-\tilde{N})+M,& u\in [\tilde{N},\frac{M(\tilde{N}+1)}{K+M}], \\ Ku, & u\ge \frac{M(\tilde{N}+1)}{K+M}. \end{array} \right. \end{aligned}$$

provided that $K\le 0$. Clearly, according to the definition of $A_{K,M}$, we have for any $K\in \mathbb {R}$, $\bar{f}_{K}(u)=Ku$ for $u\ge A_{K,M}$.

For any $r>e^{|K|\tau }\max \{A_{K,M}, \bar{N},\tilde{N}\}$, let $\bar{u}: \mathbb {R}_+\rightarrow \mathbb {R}$ be the solution of the following ODE equation:

$$\begin{aligned} \left\{ \begin{array}{ll} \bar{u}_t=\bar{f}_K(\bar{u}), & t\in \mathbb {R}_+, \\ \bar{u}(0)=r. \end{array} \right. \end{aligned}$$

Let $t^*=\inf \{t\ge 0: \bar{u}(t)=A_{K,M}\}$. Then $t^*>0$ and $\bar{u}(t)\ge A_{K,M}, \forall ~ 0\le t\le t^*$. It follows from comparison principle and the expression of $\bar{f}_K$ that $\Phi _t[r]\le \bar{u}(t)=r e^{Kt}, \forall ~ 0\le t\le t^*$. We claim that $t^*\ge \tau$. Otherwise, there exists $t^*\in (0,\tau )$ such that one of the following two cases happens:

- If $$K>0$$, then $$\bar{u}(t^*)=r e^{Kt^*}\ge r>A_{K,M}$$.
- If $$K\le 0$$, then $$\bar{u}(t^*)=r e^{Kt^*}\ge r e^{K\tau }>A_{K,M}$$.

This leads to a contradiction with the definition of $t^*$, thereby proving the claim. Therefore, we can conclude that $\Phi _\tau [r] \le \bar{u}(\tau )=r e^{K\tau }$. This, together with **(G)**, entails that $g(\cdot ,\Phi _\tau [C_r]) \subseteq C_r$ provided $Le^{K\tau }\le 1$, and hence $Q[C_r]=\mathcal {K}* g(\cdot ,\Phi _\tau [C_r]) \subseteq C_r$, $\forall r>e^{|K|\tau }\max \{A_{K,M}, \bar{N},\tilde{N}\}$.

Due to the compactness of $\Phi _\tau$ and the continuity of $\mathcal {K}*g$, we deduce that $Q[C_r]=\mathcal {K}*g(\cdot ,\Phi _\tau [C_r])$ is precompact in *C*. $\square$

### Proof of Lemma 1

#### Proof

(i) follows from the Proposition 2 and the choice of $$r^{**}$$ that (i) holds.

By **(A)**, we observe that there exists $\delta _0>0$ such that

12 $$\begin{aligned} (\delta _0+\limsup \limits _{\Vert x\Vert \rightarrow \infty } \partial _u g(x,0)) e^{(\limsup \limits _{\Vert x\Vert \rightarrow \infty }\partial _u f(x,0)+\delta _0)\tau } <1. \end{aligned}$$

Let

$$\begin{aligned} \overline{f}(x,u)=\max \big \{f(x,u),(\delta _0+\limsup \limits _{\Vert x\Vert \rightarrow \infty }\partial _u f(x,0))u\big \}\quad \text{ for } \quad (x,u)\in \mathbb {R}^N\times \mathbb {R}_+. \end{aligned}$$

and

$$\begin{aligned} \overline{g}(x,u)=\max \big \{g(x,u),(\delta _0+\limsup \limits _{\Vert x\Vert \rightarrow \infty }\partial _u g(x,0))u\big \}\quad \text{ for } \quad (x,u)\in \mathbb {R}^N\times \mathbb {R}_+. \end{aligned}$$

Then $\overline{h}\ge h$ and $\lim \limits _{\Vert x\Vert \rightarrow \infty }\overline{h}(x,u)=\overline{h}^+:=(\delta _0+\limsup \limits _{\Vert x\Vert \rightarrow \infty } \partial _u h(x,0))u$ in $C_{loc}(\mathbb {R}_+)$ for $u\in \mathbb {R}_+$, where $h=\{f,g\}$ and $\overline{h}=\{\overline{f},\overline{g}\}$. Let ${\widehat{Q}}:= \mathcal {K}*\overline{g} (\cdot ,\Phi [\tau ,\cdot ;\overline{f}])$ and $\widehat{Q}^+:= \mathcal {K}*\overline{g}^+ (\Phi ^+[\tau ,\cdot ;\overline{f}^+])$. It follows from the comparison principle that ${\widehat{Q}}[\varphi ]\ge Q[\varphi ]$ for all $\varphi \in C_+$.

(ii) follows from (12) and (Vasilyeva et al. 2016, Proposition 4.1).

(iii) By Proposition 3-(ii), we get

$$\begin{aligned} \lim \limits _{k,\Vert x\Vert \rightarrow \infty } T_{-x}\circ {\widehat{Q}}^n \circ T_x[\varphi _k]=(\widehat{Q}^+)^n[\varphi ], \quad \forall n\in \mathbb {N}, \end{aligned}$$

in *C* with $\lim \limits _{k\rightarrow \infty }||\varphi _k-\varphi ||=0$ for $(\varphi ,\varphi _k)\in C_+ \times C_+$, where $\sup \{|\varphi _k(x)|:x\in \mathbb {R}^N,k\in \mathbb {N}\}<\infty$. In particular,

$$\begin{aligned} \liminf \limits _{\Vert x\Vert \rightarrow \infty }\big [\inf \{ (\widehat{Q}^+)^n[r^{**}](0)- T_{-x}\circ {\widehat{Q}}^n \circ T_x[r^{**}](0)\}\big ]=0. \end{aligned}$$

This, combined with ${\widehat{Q}}\ge Q$, yields (iii). $\square$

### Proof of Lemma 3

#### Proof

(i) follows from Lemma 2-(i) and (Yi and Zhao 2023, Lemma 4.8-(i)).

(ii) By $\limsup \limits _{\Vert x\Vert \rightarrow \infty }\eta (x)e^{\limsup \limits _{\Vert x\Vert \rightarrow \infty }\theta (x)\tau }<1$, we see that there exists $\epsilon >0$ such that

13 $$\begin{aligned} (\limsup \limits _{\Vert x\Vert \rightarrow \infty }\eta (x)+\epsilon )e^{(\limsup \limits _{\Vert x\Vert \rightarrow \infty }\theta (x)+\epsilon )\tau }<1. \end{aligned}$$

Let

$$\begin{aligned} \overline{\eta }_\epsilon (x)=\max \{\eta (x),\limsup \limits _{\Vert x\Vert \rightarrow \infty }\eta (x)+\epsilon \},\qquad \overline{\theta }_\epsilon (x)=\max \{\theta (x),\limsup \limits _{\Vert x\Vert \rightarrow \infty }\theta (x)+\epsilon \} \end{aligned}$$

for $x\in \mathbb {R}^N$. Then $\lim \limits _{\Vert x\Vert \rightarrow \infty }\overline{\eta }_\epsilon (x)=\overline{\eta }^+_\epsilon :=\limsup \limits _{\Vert x\Vert \rightarrow \infty }\eta (x)+\epsilon$, $\lim \limits _{\Vert x\Vert \rightarrow \infty }\overline{\theta }_\epsilon (x)=\overline{\theta }^+_\epsilon :=\limsup \limits _{\Vert x\Vert \rightarrow \infty }\theta (x)+\epsilon$ and $\overline{\eta }_\epsilon (x)\ge \eta (x)\ge 0$, $\overline{\theta }_\epsilon (x)\ge \theta (x)$ for $x\in \mathbb {R}^N$. It follows from the comparison principle that $\mathcal {L}[\cdot ;\overline{\eta }_{\epsilon },\overline{\theta }_{\epsilon }]\ge \mathcal {L}$.

By (13), we find that $\lim \limits _{n\rightarrow \infty }|| (\mathcal {L}[\cdot ;\overline{\eta }^+_{\epsilon },\overline{\theta }^+_{\epsilon } ])^n[1]||_{L^\infty }=0$ due to (Vasilyeva et al. (2016), Proposition 4.1). On the other hand, applying Proposition 3-(ii) to $\mathcal {L}[\cdot ;\overline{\eta }_\epsilon ,\overline{\theta }_\epsilon ]$, we can verify asymptotic translation (i.e. **(AT)**) condition in Yi and Zhao (2023). These, combined with (Yi and Zhao 2023, Lemma 2.6), imply that $\mathcal {L}[\cdot ;\overline{\eta }_{\epsilon },\overline{\theta }_{\epsilon }]$, and hence $\mathcal {L}$ satisfies **(AC)** in (Yi and Zhao 2023, Theorem 2.5). This completes the proof. $\square$
